# Distribution update of water deer (*Hydropotes inermis*) and prediction of their potential distribution in Northeast China

**DOI:** 10.1038/s41598-023-32314-z

**Published:** 2023-04-05

**Authors:** Zongzhi Li, Romaan Hayat Khattak, Xingzhi Han, Nan Zhang, Jianping Wu, Zhensheng Liu, Liwei Teng

**Affiliations:** 1 College of Wildlife and Protected Areas, Northeast Forestry University, Harbin, 150040 China; 2 Key Laboratory of Conservation Biology, National Forestry and Grassland Administration, Harbin, 150040 China

**Keywords:** Biodiversity, Biogeography, Conservation biology, Ecological modelling

## Abstract

Human interventions have a great potential of spatially cornering and limiting species, therefore investigating the species distribution is one of the most crucial issues for managing wildlife populations and suggesting robust conservation strategies. Water deer (*Hydropotes inermis*) are widespread in China throughout history and are endemic to East Asia. However, they disappeared from Northeast China for years. We rediscovered the water deer in a previous study in Jilin Province, China. Then, we conducted further research in Northeast China to determine their distribution status, supplying fundamental data for the recovery and expansion of their population. An interview survey, line transect survey and infrared camera monitoring were carried out in some counties/cities in Northeast China from June to December 2021. The results showed that the water deer were distributed in Wuchang city of Heilongjiang Province, Changbai Korean Autonomous County, Baishan Municipal District, Ji’an city, Hunchun city, Huadian city, Antu County and Helong County of Jilin Province, Benxi Manchu Autonomous County, Huanren Manchu Autonomous County, Kuandian Manchu Autonomous County, Fengcheng city and Donggang city of Liaoning Province. The ensemble species distribution model constructed by sdm within the TSS of various models that were set as weight revealed that the potential distribution area of the water deer in the study area was 8764.66 km^2^ (28.77% of the study area). Combining recent studies concerning the distribution of water deer and the current study, we updated the distribution of wild water deer in Northeast China, which is vital for their conservation worldwide.

## Introduction

The distribution status, one of the most basic pieces of information about a species, is crucial for formulating conservation strategies for endangered species. The global species distribution pattern is shaped by multiple factors, including anthropogenic disturbance, pollution, land-use and land-cover change, overexploitation, and climate change^1,2^. The rapid expansion of human activities has dramatically changed environments worldwide, which has reshaped global biodiversity and caused well-documented shifts in the spatial distribution of wildlife^3–5^. In addition, global ecosystems and biodiversity are suffering from climate change, one of the most serious global environmental concerns—posing multiple threats to ecosystems and species^6–8^. The population and distribution of several animal and plant species have suffered dramatic declines and even extinction during recent decades due to ongoing climate change^9–14^. The distribution of species is likely to be continuously affected by the continuous expansion of human activities and increasingly frequent climate change. Therefore, it is essential to determine the current distribution pattern of species and the factors affecting their distribution.

Species distribution models (SDMs) have been widely used to predict the potential distribution (habitat suitability) of species in given areas using occurrence data of species and environmental data, particularly at large spatial and temporal scales^15–20^. Various software programs have been designed to construct SDMs, some of which performed well when using only presence-only datasets, e.g., BIOCLIM, DOMAIN and LIVES^21–23^. In comparison, others need both the presence and the absence data, e.g., generalized linear model (GLM) and generalized additive model (GAM)^22,24^. However, absence data are either difficult to obtain or very strenuous to interpret since the species database generally records only presence data^15^. Hence, several approaches were used to generate pseudoabsence data, e.g., selecting random points from the study area, which is the simplest and most widely used method in ecology^25–27^. Due to different performances between individual models and estimating uncertainty, ensemble SDMs have been recommended by several researchers, and they believe that their prediction accuracy is higher than that of single models^15,28–31^. Selecting optimal SDMs for particular species in a given area and condition is limited by many factors, e.g., species body size, environment complexity, and data resolution. Therefore, ensemble SDMs, which combine various individual models constructed with different modelling techniques, have been widely used^28,32^. Various packages that construct ensemble SDMs have been exploited to better predict the potential distribution of species, such as the packages biomod and sdm^28,33^.

Water deer (*Hydropotes inermis*) is a small deer endemic to East Asia. There are two subspecies of this deer: Chinese water deer (*H*. *i*. *inermis* Swinhoe, 1870), naturally distributed in China, and Korean water deer (*H*. *i*. *argyropus* Heude, 1884), naturally distributed in the Korean Peninsula^34–36^. The historical range of water deer was vast in China, including the Liaodong Peninsula, the North China Plain and the areas alongside the Yangtze River^37,38^. However, habitat loss and fragmentation under the pressure of climate change and anthropogenic disturbance have led to a continuous reduction in the geographical distribution area and population of wild water deer in China^39^. Hence, the deer was listed as a national key protected class II wildlife of China and as vulnerable (VU) globally by the International Union for Conservation of Nature (IUCN)^40^. The population of water deer in China was estimated at 10,000–30,000 individuals in the 1990s^35^. However, its population size is currently unclear, and it is mainly distributed in four regions that are isolated from each other, i.e., coastal area of Jiangsu Province, Zhoushan Islands of Zhejiang Province, Poyang Lake area of Jiangxi Province, and Dongting Lake area of Hunan and Hubei Provinces^35,37,41^.

Usually, it is believed that there has been no water deer population in Northeast China in recent decades^42,43^. However, studies on this area’s water deer (distribution included) have been extremely scarce. In 2017, we rediscovered the water deer population in the Baishan Musk Deer National Nature Reserve in Jilin Province of China^44^. Then, we obtained numerous reports about the distribution of water deer in Jinlin and Liaoning Provinces, and related studies were performed^45,46^. In addition, studies have shown that the water deer have spread to the far east of Russia, where there were historically no reports of water deer^47,48^. On the basis of the information we collected at present, the population of water deer in Northeast China would likely be relatively stable and healthy. However, the specific distribution and potential distribution remained unclear.

Anthropogenic disturbance and climate change have led to changes in the global environment and the distribution pattern of various habitats. In the present study, we aimed to determine the distribution pattern and potential distribution of water deer in the current environment. We carried out a field investigation in the Yalu River basin of China I to clarify the distribution of water deer in Northeast China, II to update the distribution ranges of water deer in China, combined with the current study and other studies concerning the distribution of this ungulate, and III to predict the potential distribution of water deer in the study area using the occurrence data of the deer in the current study and the environmental data. We believe that the current study could supply fundamental information for the distribution of water deer in Northeast China and even the world, which is crucial for conservation and further research on this vulnerable ungulate.

## Materials and methods

### Study area

**Figure 1:**
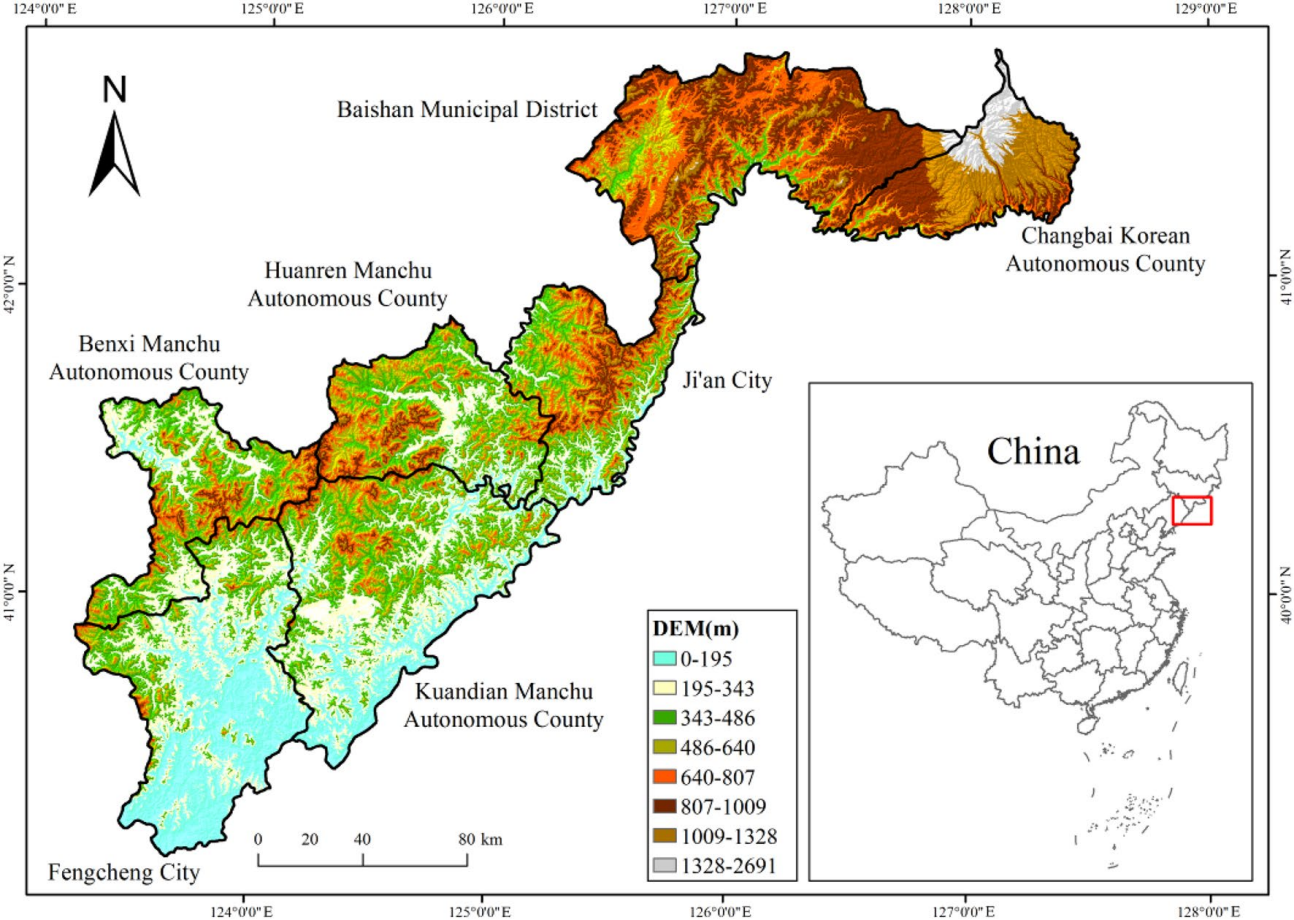
The map of the study area generated with ArcGIS 10.7 (Environmental Systems Research Institute, Inc. in Redlands, California, USA). The different colors represent different elevation of the study area, and the digital elevation model (DEM) was downloaded at https://www.gscloud.cn/.

Based on previous studies, interviews, and news reports, the study areas included some counties/cities of the Yalu River basin of China (the area is ~ 30,464.58 km^2^, Fig. 1), where the water deer were distributed throughout history. The study areas included Changbai Korean Autonomous County, Baishan Municipal District, Ji’an city in Jilin Province, Benxi Manchu Autonomous County, Huanren Manchu Autonomous County, Kuandian Manchu Autonomous County and Fengcheng city of Dandong city in Liaoning Province (Fig. 1). The geographical coordinates of the study area are E120°0′ to E123°35′, N39°59′ to N42°11′, with a north–south span of ~ 260 km and a west–east span of ~ 420 km. Baishan city belongs to the northern temperate continental monsoon climate and is the coldest area in Jilin Province. The annual average temperature is 4.6 °C, and the annual average precipitation is 883.4 mm. Dandong city has a warm temperate humid, monsoon climate; its annual average rainfall is from 800 to 1200 mm, and its annual average temperature is from 6 to 9 °C. The study area is located on the north and northwest banks of the Yalu River. The northeastern part of the study area has a relatively higher elevation, whereas the southwestern part of the area has a relatively lower elevation. Changbai Mountain is the highest part of the region, and its main peak, viz. Baiyun Peak, has an elevation of 2691 m^49^. In addition to water deer, other notable species inhabit the study area, such as Siberian musk deer (*Moschus moschiferus*), roe deer (*Capreolus pygargus*), black bear (*Ursus thibetanus*), and Eurasian lynx (*Lynx lynx*). The study area is rich in vegetation resources and diverse habitat types, including broad-leaved forest, mixed coniferous and broad-leaved forest, coniferous forest, shrub, and grassland. The study area has a developed water system adjacent to the Yalu River and several tributaries of the Yalu River, such as the Hun River, Ai River, and Cao River.

### Distribution data of water deer

The presence and absence data of water deer were collected by interview survey, random line transect survey and camera traps (Ltl 6210 MC from Ltl Acorn Electronics Co., Ltd.) monitoring. Based on the results of these investigations, the absence points of water deer were carefully selected in combination with the home range and daily activity rhythm of them. The absence points of the deer were selected from the line transects in which no individuals or their marks were found. In addition, the infrared camera locations that never captured photos/videos of the species were recorded as absence points. Certainly, these absence points were far from the known distribution of the deer, and their habitat characteristics were significantly different. The performance of some SDMs is relatively high when only the presence data of a species are available^21–23^, or they can generate pseudoabsence data using several approaches before prediction to obtain good performance^25–27^. However, these pseudoabsence data are generated based on the presence data and environment data^50–52^, which is just a principle of SDMs, and they may not be the real absence point of the species. In addition, literature or interview surveys were conducted to collect the distribution data of the deer in China, Russia, North Korea, and South Korea.

We know that it is risky for us to use the absence points identified by us in the current study because these points may not truly lack water deer. We believe that it may be more credible than the absence data generated by models, and its characteristics may be closer to those of the habitats where the water deer are not distributed. We obtained 1296 presence points and 158 absence points for water deer from the line transect survey and infrared camera monitoring. Since the body size of water deer is smaller than that of red deer (*Cervus canadensis*) and other large deer, the home range of water deer is relatively small, and there is high spatial overlap among individuals^53,54^. Hence, the presence/absence points with a distance of less than 500 m were excluded to avoid pseudoreplication. Finally, we obtained 156 presence points and 54 absence points for water deer. The presence/absence points were stored as a .shp file in ArcGIS 10.7 for further analysis.

### Collection of environment data

Based on previous research on water deer and other deer species and habitat studies on similar species^45,54,55^, the bioclimate factors (bio_1: annual mean temperature, bio_5: max temperature of the warmest month, bio_6: min temperature of the coldest month, bio_12: annual precipitation, http://www.worldclim.org/), topographical factors (elevation, slope, slope direction, https://www.gscloud.cn/), distance to the water source (National Catalogue Service for Geographic Information, https://www.webmap.cn/), vegetation factors (normalized difference vegetation index (NDVI), https://www.gscloud.cn/), and human disturbance factors (distance to road, national basic geographic information of China, http://www.ngcc.cn/) were selected to construct SDMs (Table 1). The coordinates, geographical spatial scope, and raster size of various factors were unified and stored as an .asc file using ArcGIS 10.7 for further analysis.

**Table 1:** Variance inflation factor value and data sources of selected factors.

Factors	VIF	Source
Annual precipitation	1.550	http://www.worldclim.org/
Elevation	1.804	https://www.gscloud.cn/
NDVI	1.414	https://www.gscloud.cn/
Distance to road	1.110	http://www.ngcc.cn/
Slope	1.194	https://www.gscloud.cn/
Slope direction	1.006	https://www.gscloud.cn/
Distance to water source	1.115	https://www.webmap.cn/

### Construction of SDMs

The sdm package in R was used to construct the SDMs^33^. First, the VIF (variance inflation factor) function of the usdm package was used to calculate the VIF of various factors, and the factors with a VIF value larger than 10 were excluded (Table 1)^56,57^. Bio_1, bio_5, and bio_6 were excluded. The rest of the factors were used to test the performance of various models, which were evaluated by the true skill statistic (TSS) and the area under the curve (AUC) of the receiver operating characteristic curve (ROC)^58^. Finally, boosted regression trees (brt), classification and regression trees (cart), multivariate adaptive regression splines (mars), random forest (rf), and support vector machine (svm) were selected^33^. We randomly assigned 70% of the dataset as the training dataset, and model performance was tested with the remaining 30%. The models were run for 30 replicates using a bootstrap method. Then, the ensemble function in the sdm package was used to construct ensemble SDMs within the TSS of various models that were set as weight^33^. The average TSS from the 30 replicates of the models was set as the threshold to derive presence-absence distributions from the continuous model outputs of habitat suitability^55,59,60^. Every output cell was categorized as either present (above the cut-off) or absent (below the cut-off) in ArcGIS 10.7, and we obtained the potential distribution of the water deer. Since the reliability or amount of the presence data we obtained in Russia and the Korean Peninsula was relatively lower than that in China, we predicted only the potential distribution of water deer in Northeast China.

## Results

### The distribution of water deer in Northeast China

**Figure 2:**
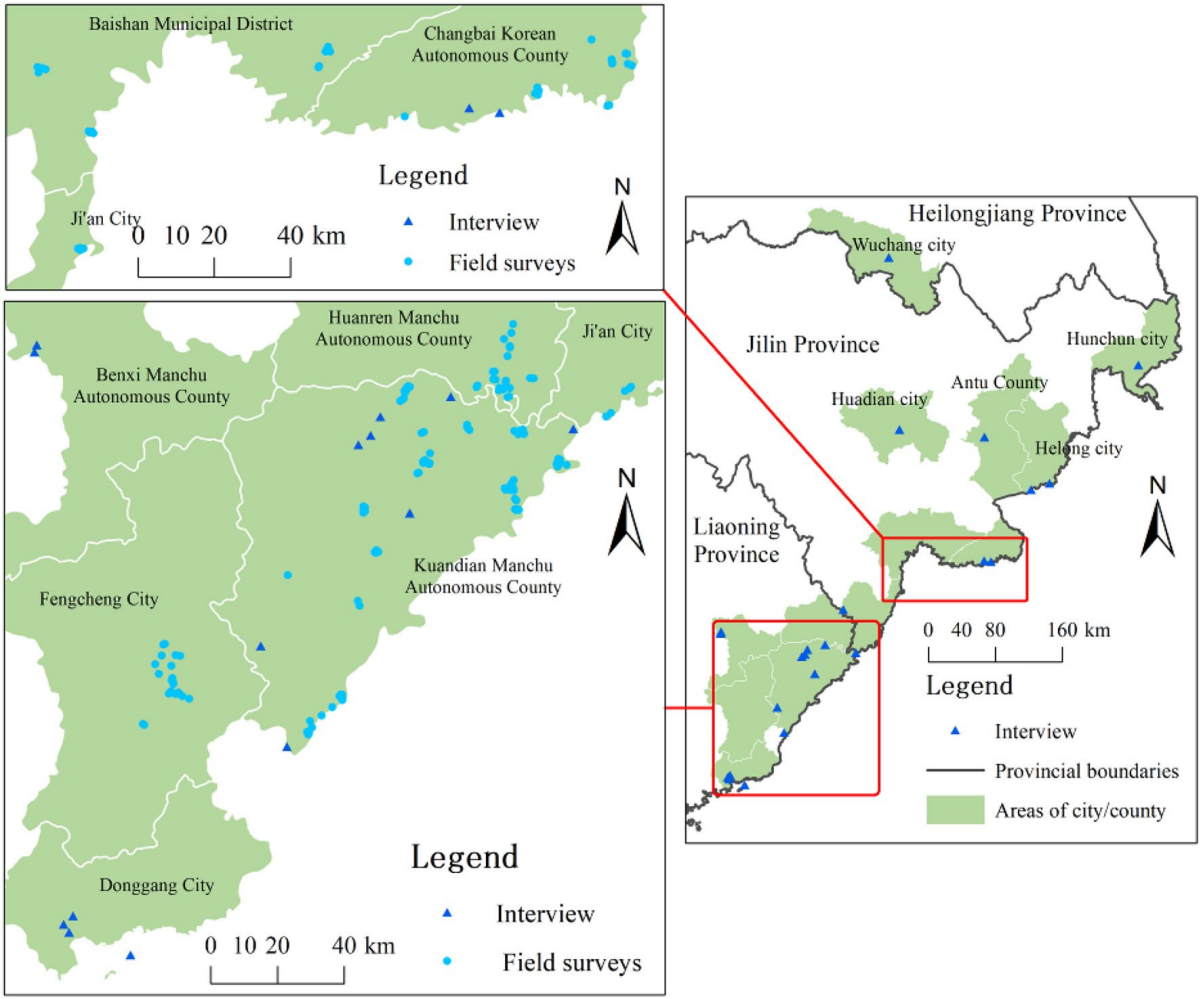
The distribution status of water deer in Northeast China based on our field surveys and interview. The circular points come from line transects survey and infrared camera monitoring, the triangular points come from interview. This map was generated with ArcGIS 10.7 (Environmental Systems Research Institute, Inc. in Redlands, California, USA. URL: https://www.esri.com/en-us/arcgis/products/arcgis-desktop/overview).

**Figure 3:**
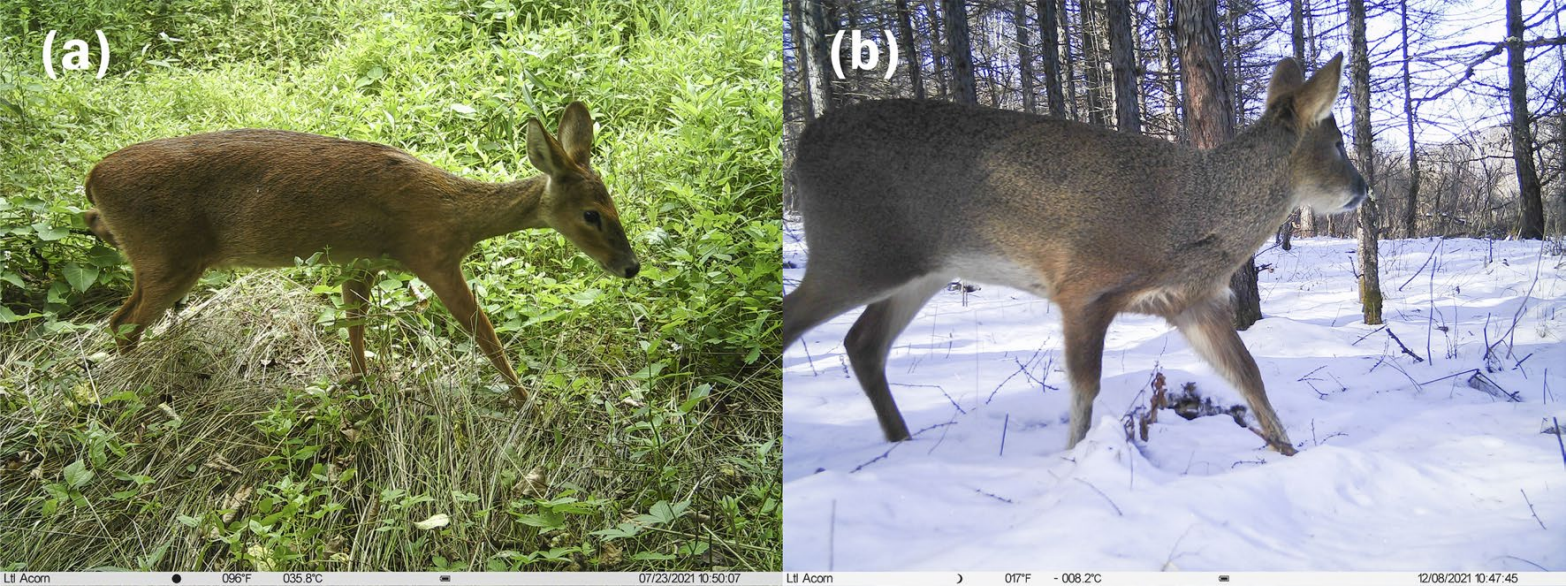
Photos of water deer captured by the infrared camera. (**a**) Photo of female water deer captured in summer. (**b**) Photo of male water deer captured in winter.

The line transects survey and infrared camera monitoring showed that water deer are distributed in Changbai Korean Autonomous County, Baishan Municipal District, and Ji’an city of Jilin Province, whereas its distribution range is relatively larger in Kuandian Manchu Autonomous County, Huanren Manchu Autonomous County, and Fengcheng city of Liaoning Province. Particularly, there is a wide distribution range of water deer in Kuandian Manchu Autonomous County, which account for almost 3/4 of the county. In addition, interview surveys and news reports indicated that water deer are distributed in Wuchang city of Heilongjiang Province; Hunchun city, Huadian city, Antu County and Helong County of Jilin Province; and Benxi Manchu Autonomous County, Donggang city of Liaoning Province (Figs. 2, 3).

**Figure 4:**
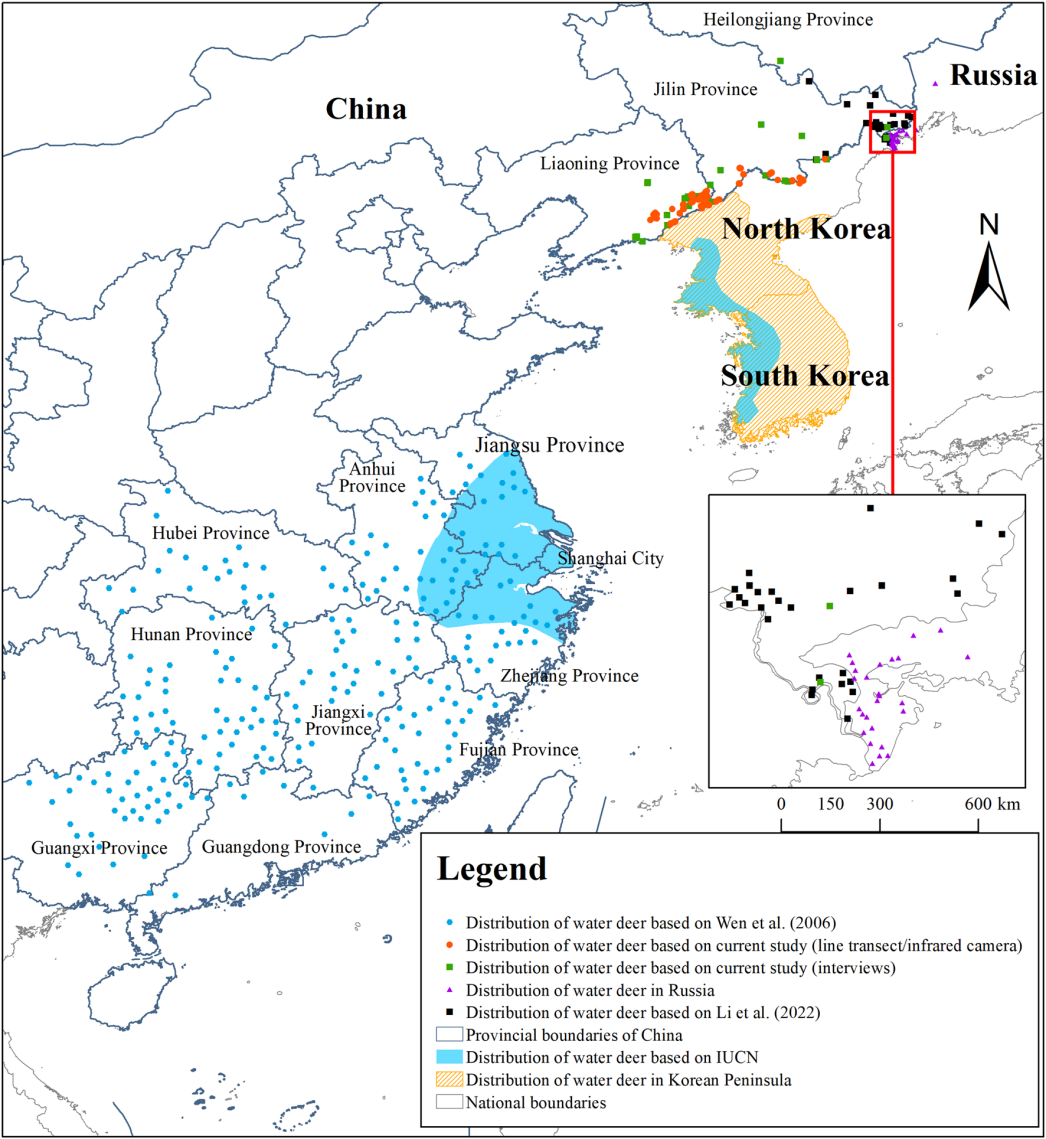
The distribution status of water deer in Northeast China and its surrounding areas. The blue points reflects the distribution of water deer in the south China which performed in 2006^62^. The orange and green points reflect their distribution in Northeast China in current study. The purple points reflects their distribution in Russia^47,48^. The black points is the distribution of water deer based on Li et al.^64^. The blue areas is their distribution based on IUCN^40^. The orange areas with diagonal reflects their current distribution in the Korean Peninsula^34^. This map was generated with ArcGIS 10.7 (Environmental Systems Research Institute, Inc. in Redlands, California, USA. URL: https://www.esri.com/en-us/arcgis/products/arcgis-desktop/overview).

We rediscovered the distribution of water deer in Jilin Province of China in 2017^44^. Darman et al. (2019) first discovered the water deer distribution in the Khasansky district of Primorsky Province, Russia^47,48^. Then, Dmitry et al. discovered the distribution of water deer in Mikhailovskiy district, Russia, which was thought to be the northernmost distribution of water deer in the world^61^. Combined with our previous and related recent studies concerning water deer^34,40,44,47,48,61–64^, we believe that it is meaningful and essential to update the distribution range of water deer. Therefore, according to recent studies, we generated a new distribution map for water deer, in which Northeast China, Russia, and the Korean Peninsula were included (Fig. 4).

### The performance of various models

All of the AUC values of various models were at least 0.84 (lowest: AUC of cart = 0.84, highest: AUC of rf = 0.95) (Table 2). The relative variable importance of the NDVI (n = 23.90%) was highest among the various factors, followed by that of elevation (n = 20.40%) and bio_12 (n = 13.00%) (Table 3). The average TSS of 30 replicates of the five models was 0.701. This threshold was used to categorize the continuous outputs of habitat suitability from ensemble SDMs as presence/absence data.

**Table 2:** The performance of various models.

Methods	AUC	COR	TSS	Deviance
brt	0.86	0.60	0.64	0.87
cart	0.84	0.65	0.64	0.98
mars	0.85	0.65	0.68	3.50
rf	0.95	0.81	0.82	0.51
svm	0.88	0.69	0.73	0.68

**Table 3:** Summary of relative variable importance of the selected factors.

Factors	Relative variable importance (%)
Annual precipitation	13.00
Elevation	20.40
NDVI	23.90
Distance to road	6.30
Slope	3.60
Slope direction	3.50
Distance to water source	3.50

### Potential distribution of water deer in Northeast China

**Figure 5:**
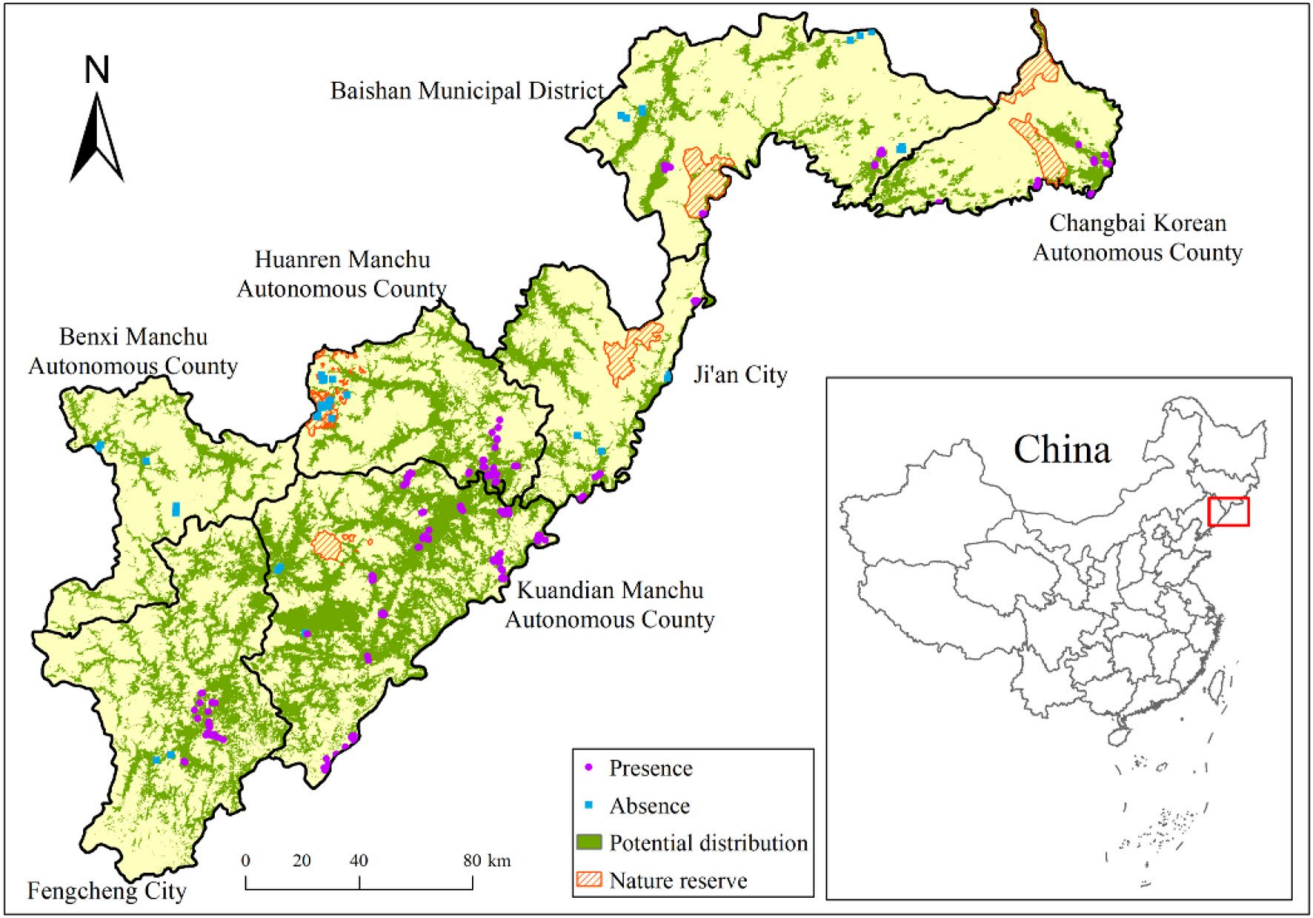
Potential distribution map of water deer generated by the ensemble SDMs and the range of nature reserves (orange areas with diagonal) in Northeast China. The purple points are the presence location of water deer, and the blue points are the absence location of the deer which was defined by current study. This map was generated with ArcGIS 10.7 (Environmental Systems Research Institute, Inc. in Redlands, California, USA. URL: https://www.esri.com/en-us/arcgis/products/arcgis-desktop/overview).

The results revealed that the whole potential distribution area of water deer in the study area was 8764.66 km^2^ (28.77%). Its potential distribution area was largest in Kuandian Manchu Autonomous County, reaching 3078.46 km^2^ (49.23%). In contrast, it was the smallest in Changbai Korean Autonomous County, with only 494.80 km^2^ (19.83%) (Fig. 5, Table 4).

**Table 4:** Potential distribution area of water deer in various cities/counties in Northeast China.

County/city	Potential distribution (%)	Area (km^2^)
Baishan Municipal District	15.29	875.00
Changbai Korean Autonomous County	19.83	494.80
Ji’an city	23.30	771.39
Huanren Manchu Autonomous County	30.61	1092.22
Benxi Manchu Autonomous County	18.33	613.67
Kuandian Manchu Autonomous County	49.23	3078.46
Fengcheng city	32.02	1844.47
Whole	28.77	8764.66

## Discussion

In the current study, a line transects survey, infrared camera monitoring, an interview survey, and a literature survey were employed. The results showed that the water deer were distributed in several counties/cities of the Yalu River basin of China. The distribution range of water deer in Northeast China was vaster than previously thought^47,48,61^. In addition, combining our results with related research on the deer, we updated the distribution range of its natural population in China, which may indicate its global distribution should be updated. Water deer presence/absence data in Northeast China were employed to predict its potential distribution. There was a considerable potential distribution of the deer in the study area, which is a crucial area for further conservation and study in China. It should be noted that the potential distribution range of water deer in various counties/cities was different.

Throughout history, the distribution of water deer in China was vast, and its range reached from N45°42′ (Harbin, Heilongjiang) to N18°24′ (Lingshui, Hainan) and from E97°6′′ (Changdu, Tibet) to 122°24′ (Shengsi, Zhejiang). However, in the 1980s, its distribution shrank from N34°6′ (Shuyang, Jiangsu) to N21°30′ (Dianbai, Guangdong) and east of E107°6′ (Tian’e, Guangxi)^62^. The population decline in China in the past ten thousand years was due to the decrease in its living space caused by climate change, agricultural production, human disturbance, and a decline of shallows^65–69^. Moreover, the extreme decline in water deer in recent decades was caused by habitat degradation, habitat loss, and overhunting, particularly in the south of the Yangtze River^70–73^. Then, the water deer was introduced to England, and the escaped individuals formed a wild population^74,75^, which accounts for more than 40% of the wild population in the world^76^. Previous research indicated that the population of water deer in England could supply support for the population recovery of the deer in China^77^. Our study suggested that the distribution and population size of water deer in Northeast China are relatively large, and there may be no need to introduce the species from other places to recover the population. Fortunately, due to the Chinese government's strict conservation efforts and policies, many forests in China have been recovering in recent decades^78^, which provides a foundation for the population recovery of water deer.

In recent years, water deer have spread and ranged in Russia’s far east, forming healthy and stable populations^47,48,61^. The spread of the deer may be caused by habitat transformation under the effects of anthropogenic disturbance and climate change. In the most recent years, two studies focused on the distribution, habitat suitability, and dispersal routes of water deer among Northeast China, the far east of Russia, and the Korean Peninsula, while both were relatively lacking in the ranges of field investigation^64,79^. Their field investigation was lacking in Liaoning Province, China, where our results revealed that the distribution of the deer was vastest. Their habitat suitability of the deer in their study area was almost opposite^79^, which may be caused by their lack of occurrence data of the species in Liaoning. Hence, the possible dispersal routes of water deer obtained by them did not match the distribution pattern in our study^79^, which demonstrated the importance of obtaining adequate presence data and updating the distribution of the species in a comprehensive study on them.

The AUC in multiple replicates of the ensemble SDMs was relatively high (from 0.84 to 0.95), revealing that the simulated potential distribution of water deer in Northeast China is reliable, which has important guiding significance for the future conservation area designation of the species. The potential distribution area of the water deer was relatively scattered (Fig. 5), making it difficult to spread and communicate among the populations in the future. On the one hand, this may be caused by the difference in altitude and vegetation types of various habitat patches in the study area since the NDVI and elevation are crucial for the habitat of water deer (Table 3). On the other hand, roads in the study area have contributed to the dispersion of potential distribution areas of water deer to a certain extent. Based on observations in our fieldwork, many traces of water deer were found in areas close to roads or residents. Roads pose potential threats to their lives, as water deer roaming or spreading across roads are more likely to be struck and killed by cars. In South Korea, roadkill is an important factor leading to the death of water deer^80,81^. During our interviews, we also learned that there were many roadkills of water deer in Northeast China.

A total of 50.52% of the total occurrence points were collected from Kuandian Manchu Autonomous County, reflecting a large water deer population in this area. Therefore, Kuandian Manchu Autonomous County may be the most important region to maintain the stability of the water deer population in Northeast China. The ensemble SDMs revealed an enormous potential distribution of water deer in the study area (8764.66 km^2^, 28.77%, Fig. 5, Table 4). Among them, the area of the potential distribution area and the proportion of the total potential distribution area in Kuandian Manchu Autonomous County were the largest (3078.46 km^2^, 35.12%, Fig. 5, Table 4). However, the potential distribution area of water deer in protected areas in the study area was deficient (Fig. 5). Moreover, there were no protected areas in the main potential distribution area of the species, let alone the protected area specializing in the conservation of water deer (Fig. 5). Protected area with a distribution of water deer is scarce in China^54,82,83^. Protected areas are considered the cornerstones of biodiversity conservation^84–86^. According to the habitat characteristics of water deer, key conservation efforts should be carried out in their potential distribution as soon as possible. In other words, establishing a special reserve to protect the deer population in Northeast China is very important and necessary.

The relative variable importance of the NDVI (23.90%) was highest among various factors, reflecting an essential role of vegetation in the habitat of water deer. The occurrence points of the deer were found in the habitats of grass/shrub most frequently, which possess relatively good vegetation conditions and could supply favourable shelter as well as adequate/palatable food for them, and it is similar to the studies on the species in both Northeast and South China^45,54,82^. Water deer prefer grass/shrub habitats with relatively high hiding levels and good vegetation cover^45,54^. Hence, they also prefer cropland. Moreover, the deer have caused certain damage to local crops. Fabaceae account for a high proportion of the food of water deer^63^, and we found that they consumed a large amount of soybean (Glycine max) leaves in the cropland. In addition, the leaves of sweet potato (*Ipomoea batatas*) are their favourite food, and they even dig sweet potato out with their hooves. All of these croplands were close to settlements or roads, suggesting that the deer may not be afraid of humans. The relative variable importance of distance to the road (6.30%) was relatively lower than that of the other three factors, which could also prove this to some extent (Table 3).

Following the NDVI, the relative variable importance of elevation ranked second with a proportion of 20.40%. The elevation affected the distribution of different plant species in our study area. There were more grass/shrub species distributed in places with lower elevations, whereas there were more tree species distributed in places with higher elevations. Since elevation is important for the habitat selection of water deer^45^ and its relation with the distribution of plants, we summarized the typical habitats of the deer in Northeast China: 1 grassland/cropland habitat: grassland with a slope of less than 30° in the middle and low slopes and cropland of soybean, sweet potato, peanut (*Arachis hypogaea*), and corn (*Zea mays*); 2 shrub/orchard habitat: shrubs with a slope of less than 30° in the middle and low slopes and orchards of filbert (*Corylus* spp.), *Pinus koraiensis* sapling, and fruits; and 3 larch (*Larix gmelinii*) habitats: larch forest habitat with a slope of less than 30° and a canopy closure not higher than 80% in the middle and upper slopes. There are a few herbs/shrubs under the forest, the forest area is not large, and there are overlapping areas with grassland/shrub habitats. This summary of the habitat of water deer may supply guidance for conservation and future research. This summary is just our speculation and requires a future careful comparison. We will record detailed information on the habitat characteristics in different counties/cities in future studies, aiming to compare their habitat among areas and summarize their habitat with sufficient data.

The current study could not determine whether the water deer in Northeast China were remaining populations or whether they spread from the Korean Peninsula. Further study on the cross-border activity pattern and movement corridors of water deer between China and the Korean Peninsula may help to solve this issue. We know that Chinese water deer and Korean water deer are naturally distributed in China and the Korean Peninsula, respectively^34–36^. However, recent research has shown that there are two subspecies of water deer in South Korea^87^; therefore, the subspecies belonging to the water deer in Northeast China could not be used to distinguish whether they were local populations or whether they spread from the Korean Peninsula. However, the subspecies of the deer in Northeast China remains unclear and needs more research in the future.

We believe there may have been populations of water deer in Northeast China for a long time, and perhaps they never disappeared, but related research is lacking. On the one hand, the lack of research on this species may be due to its relatively lower economic value than that of sika deer (*Cervus nippon*)^88,89^. In addition, the mammals in our study area other than the water deer are musk deer (*Moschus spp.*), black bears, etc., which are more endangered or have a larger influence on humans, so there is more research on them^55,90^. Hence, little is known about the distribution and population status of water deer in Northeast China in recent years^45^. On the other hand, the locals could mistake the water deer for musk deer or roe deer because their body size and fur colour are similar^41,91^. In our investigation, if we showed a photo of water deer to locals, they were pretty sure that the species they saw was water deer, but they still called it “musk deer” or “roe deer”. In addition, the spread of water deer from the Korean Peninsula has occurred^79^. When the Yalu River freezes in winter, water deer can easily spread from North Korea to China over the ice, which locals have witnessed many times. Moreover, water deer are skilled in swimming; their swimming distance can be 2 kms or more. In the Zhoushan Archipelago of China, water deer always spread by swimming among islands^53,73,92,93^. In summary, we believe that the deer in Northeast China may have always been, and at the same time, the species may have also spread from the Korean Peninsula to China through the Yalu River.

## Conclusion

In the current study, we investigated the distribution of water deer in Northeast China and found a large distribution and population of the species alongside the Yalu River in China. We updated the distribution map of water deer in Northeast China and its surrounding areas, reflecting the distribution range of the species in Russia, the Korean Peninsula, and China. Finally, we predicted the potential water deer distribution in Northeast China. The results showed a large potential distribution area of the species in the study area, which is a good foundation for their population recovery.

## Data Availability

The raw data supporting the conclusions of this article are stored in College of Wildlife and Protected Areas, Northeast Forestry University and it will be made available by the corresponding author without undue reservation.

## References

[1] Benítez-López A, Santini L, Schipper AM, Busana M, Huijbregts MA (2019). Intact but empty forests? Patterns of hunting-induced mammal defaunation in the tropics. PLoS Biol..

[2] Butchart SH (2010). Global biodiversity: indicators of recent declines. Science.

[3] Boivin NL (2016). Ecological consequences of human niche construction: Examining long-term anthropogenic shaping of global species distributions. Proc. Natl. Acad. Sci. U.S.A..

[4] Hale R, Swearer SE (2016). Ecological traps: Current evidence and future directions. Proc. R. Soc. B Biol. Sci..

[5] Gaynor KM, Hojnowski CE, Carter NH, Brashares JS (2018). The influence of human disturbance on wildlife nocturnality. Science.

[6] Ebrahimi E, Ranjbaran Y, Sayahnia R, Ahmadzadeh F (2022). Assessing the climate change effects on the distribution pattern of the Azerbaijan Mountain Newt (*Neurergus crocatus*). Ecol. Complex..

[7] Sánchez-Fernández D, Galassi DM, Wynne JJ, Cardoso P, Mammola S (2021). Don’t forget subterranean ecosystems in climate change agendas. Nat. Clim. Chang..

[8] Bellard C, Bertelsmeier C, Leadley P, Thuiller W, Courchamp F (2012). Impacts of climate change on the future of biodiversity. Ecol. Lett..

[9] Thomas CD, Franco AM, Hill JK (2006). Range retractions and extinction in the face of climate warming. Trends Ecol. Evol..

[10] Sekercioglu CH, Schneider SH, Fay JP, Loarie SR (2008). Climate change, elevational range shifts, and bird extinctions. Conserv. Biol..

[11] Sanderson FJ, Donald PF, Pain DJ, Burfield IJ, Van Bommel FP (2006). Long-term population declines in Afro-Palearctic migrant birds. Biol. Cons..

[12] Williams SE, Shoo LP, Isaac JL, Hoffmann AA, Langham G (2008). Towards an integrated framework for assessing the vulnerability of species to climate change. PLoS Biol..

[13] Bates AE (2014). Defining and observing stages of climate-mediated range shifts in marine systems. Glob. Environ. Chang..

[14] Malcolm JR, Liu C, Neilson RP, Hansen L, Hannah L (2006). Global warming and extinctions of endemic species from biodiversity hotspots. Conserv. Biol..

[15] Liu C, Newell G, White M (2019). The effect of sample size on the accuracy of species distribution models: Considering both presences and pseudo-absences or background sites. Ecography.

[16] Aarts G, Fieberg J, Matthiopoulos J (2012). Comparative interpretation of count, presence–absence and point methods for species distribution models. Methods Ecol. Evol..

[17] Kafash A (2018). Climate change produces winners and losers: Differential responses of amphibians in mountain forests of the Near East. Glob. Ecol. Conserv..

[18] Anderson RP (2013). A framework for using niche models to estimate impacts of climate change on species distributions. Ann. N. Y. Acad. Sci..

[19] Elith J, Leathwick JR (2009). Species distribution models: Ecological explanation and prediction across space and time. Annu. Rev. Ecol. Evol. Syst..

[20] Guisan A, Thuiller W (2005). Predicting species distribution: Offering more than simple habitat models. Ecol. Lett..

[21] Li J, Hilbert DW (2008). LIVES: a new habitat modelling technique for predicting the distribution of species’ occurrences using presence-only data based on limiting factor theory. Biodivers. Conserv..

[22] Robertson M, Caithness N, Villet M (2001). A PCA-based modelling technique for predicting environmental suitability for organisms from presence records. Divers. Distrib..

[23] Booth TH, Nix HA, Busby JR, Hutchinson MF (2014). BIOCLIM: The first species distribution modelling package, its early applications and relevance to most current MAXENT studies. Divers. Distrib..

[24] Guisan A, Edwards TC, Hastie T (2002). Generalized linear and generalized additive models in studies of species distributions: Setting the scene. Ecol. Model..

[25] Stockwell D (1999). The GARP modelling system: Problems and solutions to automated spatial prediction. Int. J. Geogr. Inf. Sci..

[26] Lobo JM, Tognelli MF (2011). Exploring the effects of quantity and location of pseudo-absences and sampling biases on the performance of distribution models with limited point occurrence data. J. Nat. Conserv..

[27] Phillips SJ (2009). Sample selection bias and presence-only distribution models: Implications for background and pseudo-absence data. Ecol. Appl..

[28] Thuiller W, Lafourcade B, Engler R, Araújo MB (2009). BIOMOD–a platform for ensemble forecasting of species distributions. Ecography.

[29] Araújo MB, New M (2007). Ensemble forecasting of species distributions. Trends Ecol. Evol..

[30] Marmion M, Luoto M, Heikkinen RK, Thuiller W (2009). The performance of state-of-the-art modelling techniques depends on geographical distribution of species. Ecol. Model..

[31] Comte L, Grenouillet G (2013). Species distribution modelling and imperfect detection: Comparing occupancy versus consensus methods. Divers. Distrib..

[32] Strubbe D, Jackson H, Groombridge J, Matthysen E (2015). Invasion success of a global avian invader is explained by within-taxon niche structure and association with humans in the native range. Divers. Distrib..

[33] Naimi B, Araújo MB (2016). sdm: A reproducible and extensible R platform for species distribution modelling. Ecography.

[34] Jo Y-S, Baccus JT, Koprowski JL (2018). Mammals of Korea: A review of their taxonomy, distribution and conservation status. Zootaxa.

[35] Geist, V. *Deer of the World: Their Evolution, Behaviour, and Ecology*. (Stackpole Books, 1998).

[36] Allen, G. M. *The Mammals of China and Mongolia*. (American Museum of Natural History, 1938).

[37] Ohtaishi, N. & Sheng, H. *Deer of China: Biology and Management: Proceedings of the International Symposium on Deer of China, Held in Shanghai, China* (Elsevier, 1993).

[38] Hu J, Fang SG, Wan QH (2006). Genetic diversity of Chinese water deer (Hydropotes inermis inermis): Implications for conservation. Biochem. Genet..

[39] Hongfa X, Xiangzhong Z, Houji L (2006). Impact of human activities and habitat changes on distribution of chinese water deer along the coast arsa in northern Jiangsu. Acta Theriol. Sin..

[40] Harris, R. B. & Duckworth, J. W. Hydropotes inermis. The IUCN Red List of Threatened Species 2015: e.T10329A22163569. https://dx.doi.org/10.2305/IUCN.UK.2015-2.RLTS.T10329A22163569.en. Accessed on 12 May 2022. (2015).

[41] Sheng, H. *Deer of China*. (East China Normal University Press, 1992).

[42] Institute of Zoology, C. A. O. S. *Northeast Animal Survey Report*. (Science Press, 1958).

[43] Ma, F. & Zhang, J. *Survey on Key Terrestrial Wildlife Resources in China*. (China Forestry Publishing House, 2009).

[44] Li Z (2019). The rediscovery of water deer (Hydropotes inermis) in Jilin province Chinese. J. Zool..

[45] Li Z, Liu Z, Mi S, Wu J, Teng L (2021). Habitat selection of the Chinese water deer at Baishan Musk Deer Natural Reserve in spring and summer. Acta Ecol. Sin..

[46] Li Z (2020). The complete mitochondrial genome of water deer in Liaoning China. Mitochondrial DNA Part B..

[47] Darman YA, Storozhuk V, Sedash G (2019). Hydropotes inermis (Cervidae), a new species for the Russian fauna registered in the land of Leopard National Park (Russia). Nat. Conserv. Res..

[48] 48Darman, Y. & Sedash, G. A. Korean water deer (Hydropotes inermis argyropus Heude, 1884): General outline for enlisting into the Red Data Book of Russian Federation. *Rare Species of Biota*, 35–40 (2020).

[49] Zhao, Z. *Fauna of Rare and Endangered Species of Vertebrates of Northeast China*. (China Forestry Publishing House, 1999).

[50] Zaniewski AE, Lehmann A, Overton JM (2002). Predicting species spatial distributions using presence-only data: A case study of native New Zealand ferns. Ecol. Model..

[51] Engler R, Guisan A, Rechsteiner L (2004). An improved approach for predicting the distribution of rare and endangered species from occurrence and pseudo-absence data. J. Appl. Ecol..

[52] Lobo JM, Jiménez-Valverde A, Hortal J (2010). The uncertain nature of absences and their importance in species distribution modelling. Ecography.

[53] He, X. *Spatial Behavioural Ecology of the Chinese Water Deer*. Doctor’s thesis, East China Normal University, (2013).

[54] Zhang, N. *Home Range and Habitat Selection of Chinese Water Deer (Hydropotes inermis) After Release in Poyang Lake During the Dry Season*. Master's thesis, Jiangxi Normal University, (2019).

[55] Zhang C (2022). Identification of conservation priority areas and a protection network for the siberian musk deer (*Moschus moschiferus* L.) in Northeast China. Animals.

[56] Dormann CF (2013). Collinearity: A review of methods to deal with it and a simulation study evaluating their performance. Ecography.

[57] Naimi B, Hamm NA, Groen TA, Skidmore AK, Toxopeus AG (2014). Where is positional uncertainty a problem for species distribution modelling?. Ecography.

[58] Allouche O, Tsoar A, Kadmon R (2006). Assessing the accuracy of species distribution models: Prevalence, kappa and the true skill statistic (TSS). J. Appl. Ecol..

[59] Coetzee BW, Robertson MP, Erasmus BF, Van Rensburg BJ, Thuiller W (2009). Ensemble models predict Important Bird Areas in southern Africa will become less effective for conserving endemic birds under climate change. Glob. Ecol. Biogeogr..

[60] Yang L, Shi KC, Ma C, Ren GP, Fan PF (2021). Mechanisms underlying altitudinal and horizontal range contraction: The western black crested gibbon. J. Biogeogr..

[61] Belyaev DA, Jo Y-S (2021). Northernmost finding and further information on water deer Hydropotes inermis in Primorskiy Krai, Russia. Mammalia.

[62] Wen, H. & Wen, R. *The Change of the Plant and Animal in China During Different Historical Period*. (Chongqing Press, 2006).

[63] Huang, Y. *Food-Habits of Chinese Water Deer (Hydropotes inermis) in Poyang Lake Area*. Master's thesis, Jiangxi Normal University, (2016).

[64] Li Y (2022). Northward range expansion of water deer in Northeast Asia: Direct evidence and management implications. Animals.

[65] Huang Z, Zhang W (2004). Climate fluctuation and natural disasters during historical periods in tropics of China. J. Nat. Disasters..

[66] Fang, Y. Mammalian fauna of Jiangsu since Pliocene. *Southeast Cult.* 33–40 (2000).

[67] Zou, Y. A Brief Introduction to the Changes of Lakes and Marshes in the Great Plains of North China in the Historical Period. *Hist. Geogr.* 1–33 (1987).

[68] Wang, Q. Water deer and Paleo-environmental changes in Liaodong Peninsula. *Archaeol. Cult. Relics*, 28–34 (1999).

[69] Hou Y, Zhu Y (2000). Important climatic events showed by historical records from middle and lower reach plain of the yellow river during 5–2.7ka and their environmental significance. Mar. Geol. Quat. Geol..

[70] Yang D, Jiang Z, Ma J, Hu H, Li P (2005). Causes of endangerment or extinction of some mammals and its elevance to the reintroduction of Père David’s deer in the Dongting Lake drainage area. Biodiv. Sci..

[71] Ohtaishi N, Gao Y (1990). A review of the distribution of all species of deer (Tragulidae, Moschidae and Cervidae) in China. Mammal Rev..

[72] Cooke, A. S. & Farrell, L. *Chinese Water Deer*. (Mammal Society and The British Deer Society, 1998).

[73] Chen, M. *Genetic Diversity in and Conservation Strategy Considerations for the Chinese Water Deer (Hydropotes inermis)*. Doctor's thesis, East China Normal University, (2006).

[74] Whitehead, G. K. *The Deer of Great Britain and Ireland: An Account of Their History, Status and Distribution*. (Routledge and Kegan Paul London, 1964).

[75] Firter, R. S. R. *The Ark in Our Midst*. (Collins, 1959).

[76] Putman R (2021). Conservation genetics of native and European-introduced Chinese water deer (*Hydropotes inermis*). Zool. J. Linn. Soc..

[77] Zhongming, Z. & Wei, L. *Chinese Water Deer Introduced to UK May be Valuable to Restoring Numbers in Asia*, http://resp.llas.ac.cn/C666/handle/2XK7JSWQ/306333 (2020).

[78] Viña A, McConnell WJ, Yang H, Xu Z, Liu J (2016). Effects of conservation policy on China’s forest recovery. Sci. Adv..

[79] Li Y (2022). Prediction of range expansion and estimation of dispersal routes of water deer (*Hydropotes inermis*) in the transboundary region between China, the Russian Far East and the Korean Peninsula. PLoS ONE.

[80] Kim M, Park H, Lee S (2021). Analysis of Roadkill on the Korean Expressways from 2004 to 2019. Int. J. Environ. Res. Public Health..

[81] Kim W, Hong SH (2021). An empirical analysis on factors affecting water deer roadkills in Korea. KSCE J. Civ. Eng..

[82] Zhang E, Teng L, Wu Y (2006). Habitat suitability evaluation for the Chinese water deer (Hydropotes inermis) in Yancheng Nature Reserve China. Acta Theriol. Sin..

[83] Zhang X, Zhang E (2002). Distribution pattern of Hydropotes inermis in various habitats in Jiangsu Dafeng Pere David's deer state nature reserve. Sichuan J. Zool..

[84] Bruner AG, Gullison RE, Rice RE, Da Fonseca GA (2001). Effectiveness of parks in protecting tropical biodiversity. Science.

[85] Pimm SL, Lawton JH (1998). Planning for biodiversity. Science.

[86] Wright, R. G. *National Parks and Protected Areas: Their Role in Environmental Protection* (Blackwell Science Ltd., 1996).

[87] Kim HR, Kim EK, Jeon MG, Park YC (2015). Intraspecific phylogeny of the Korean Water Deer, hydropotes inermis argyropus (Artiodactyla, Cervidae). Anim. Syst. Evol. Divers..

[88] Nentwig W, Kühnel E, Bacher S (2010). A generic impact-scoring system applied to alien mammals in Europe. Conserv. Biol..

[89] Kumschick S (2015). Comparing impacts of alien plants and animals in Europe using a standard scoring system. J. Appl. Ecol..

[90] Yang L (2019). Potential distribution and conservation priority areas of five species in Northeast China. Acta Ecol. Sin..

[91] Wen R (2006). The differentiation between She (Moschus) and Zhang (Hydropotes inermis) in China during the historical times. J. Chin. Hist. Geogr..

[92] Sheng H, Lu H (1984). A preliminary study on the river deer population of Zhoushan island and adjacent islets. Acta Theriol. Sin..

[93] Guo G, Zhang E (2002). The distribution of the Chinese water deer (Hydropotes inermis) in Zhoushan Archipelago, Zhejiang province China. Acta Theriol. Sin..

